# Supplementary material for: Cell-free tumor DNA, CA125 and HE4 for the objective assessment of tumor burden in patients with advanced high-grade serous ovarian cancer
Source: PLoS One. 2022 Feb 7;17(2):e0262770. doi: 10.1371/journal.pone.0262770 (PMC8820624; doi:10.1371/journal.pone.0262770)
Supplement: S1 Fig — A: Serum CA125 in dependency of tumor burden (debulking status after surgery); R status- residual disease after surgery; D0 baseline; D4 day 4 after surgery; D10 day 10 after surgery; p-values report comparison between TR0 and TR>0; n.s.- not significant B: Serum HE4 in dependency of tumor burden (debulking status after surgery); R status- residual disease after surgery; D0 baseline; D4 day 4 after surgery; D10 day 10 after surgery; p-values report comparison between TR0 and TR>0; n.s.- not significantC: Ascites CA125 in dependency of tumor burden (debulking status after surgery); R status- residual disease after surgery; D0- baseline; D4 -day 4 after surgery; D10- day 10 after surgery; p-values report comparison between TR0 and TR>0; n.s.- not significantD: Ascites HE4 in dependency of tumor burden (debulking status after surgery); R status- residual disease after surgery; D0 baseline; D4 day 4 after surgery; D10 day 10 after surgery; p-values report comparison between TR0 and TR>0; n.s.- not significant. (DOCX) [file pone.0262770.s001.docx]

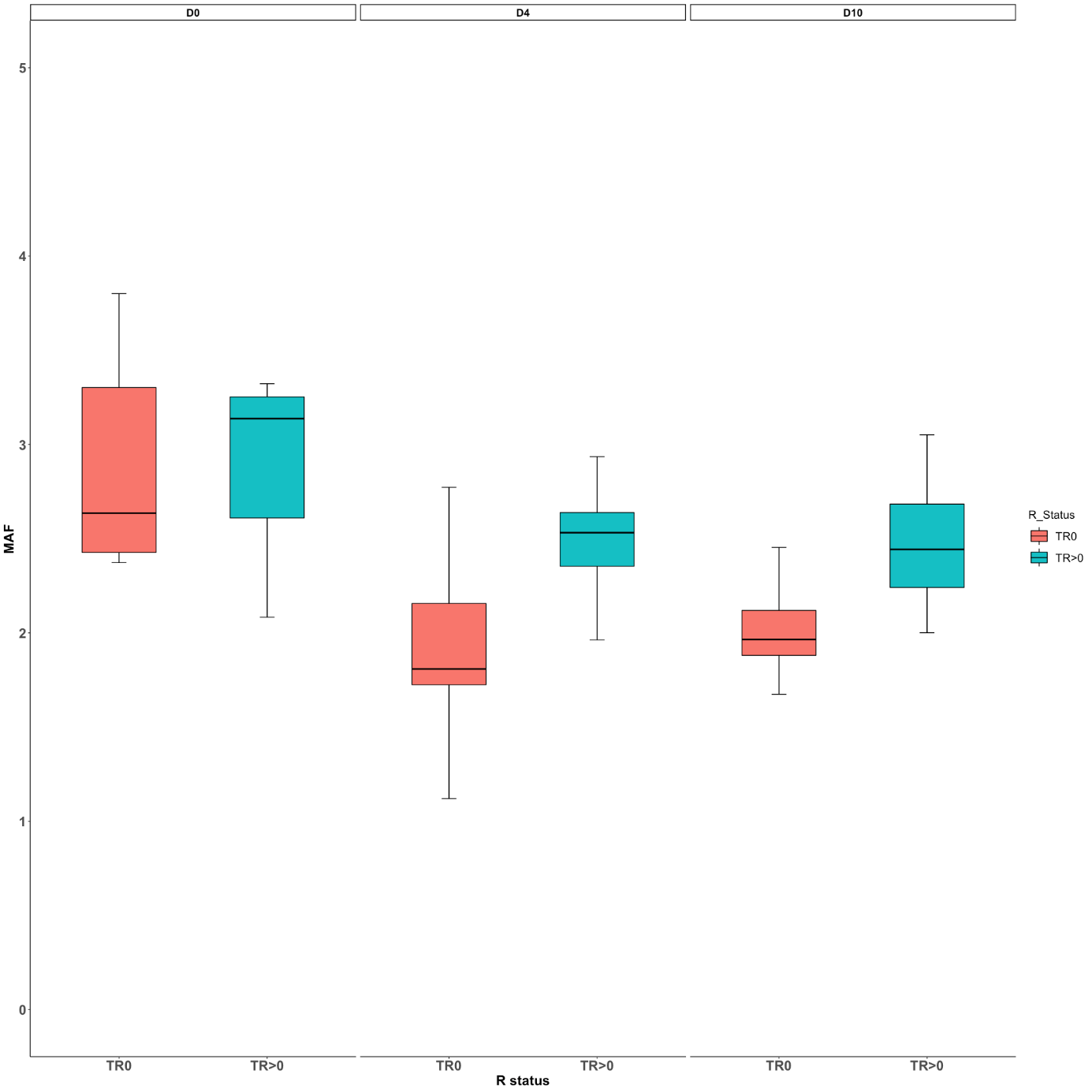


Δlog serum CA125

p=0.015

p=0.024

n.s.

Supplement 1 Figure A: **Serum CA125 in dependency of tumor burden** (debulking status after surgery); R status- residual disease after surgery; D0 baseline; D4 day 4 after surgery; D10 day 10 after surgery; p-values report comparison between TR0 and TR>0; n.s.- not significant


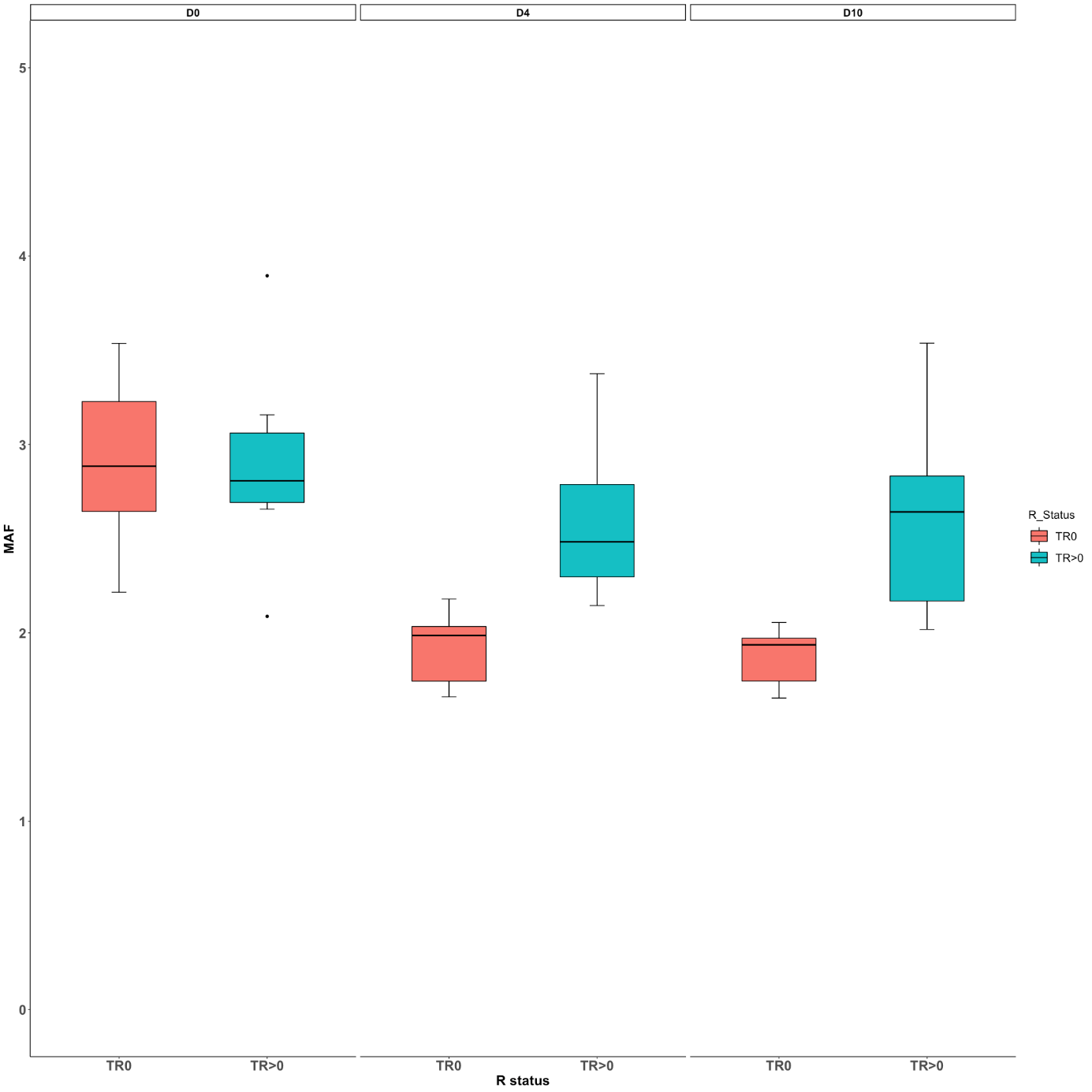


Δlog serum HE4

p=0.000

p=0.000

n.s.

Supplement 1 Figure B: **Serum HE4 in dependency of tumor burden** (debulking status after surgery); R status- residual disease after surgery; D0 baseline; D4 day 4 after surgery; D10 day 10 after surgery; p-values report comparison between TR0 and TR>0; n.s.- not significant


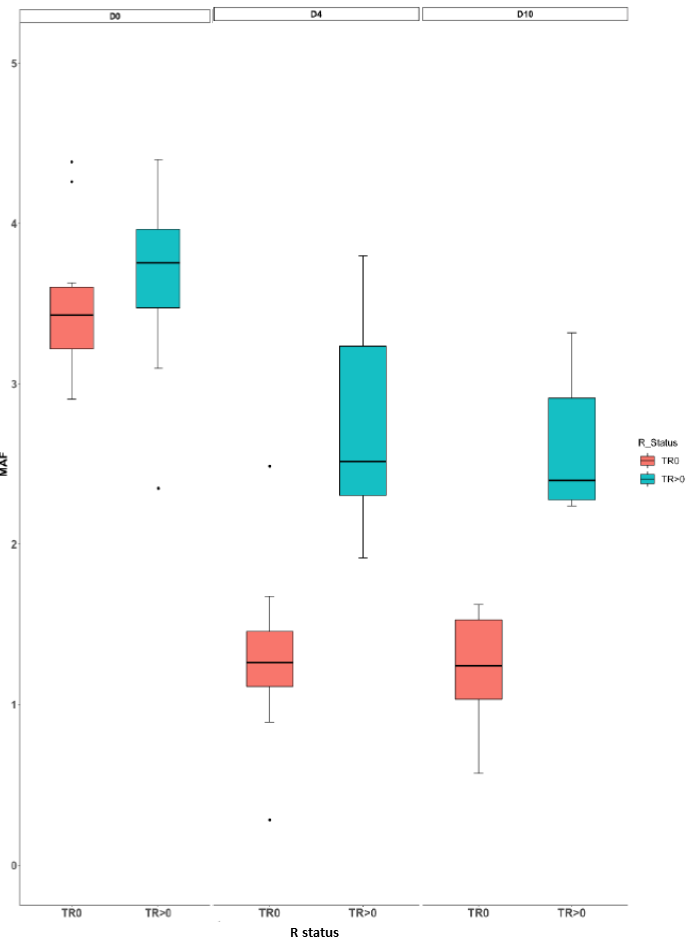


n.s.

p=0.003

p=0.000

Δlog ascites CA125

Supplement 1 Figure C: **Ascites CA125 in dependency of tumor burden** (debulking status after surgery); R status- residual disease after surgery; D0- baseline; D4 -day 4 after surgery; D10- day 10 after surgery; p-values report comparison between TR0 and TR>0; n.s.- not significant


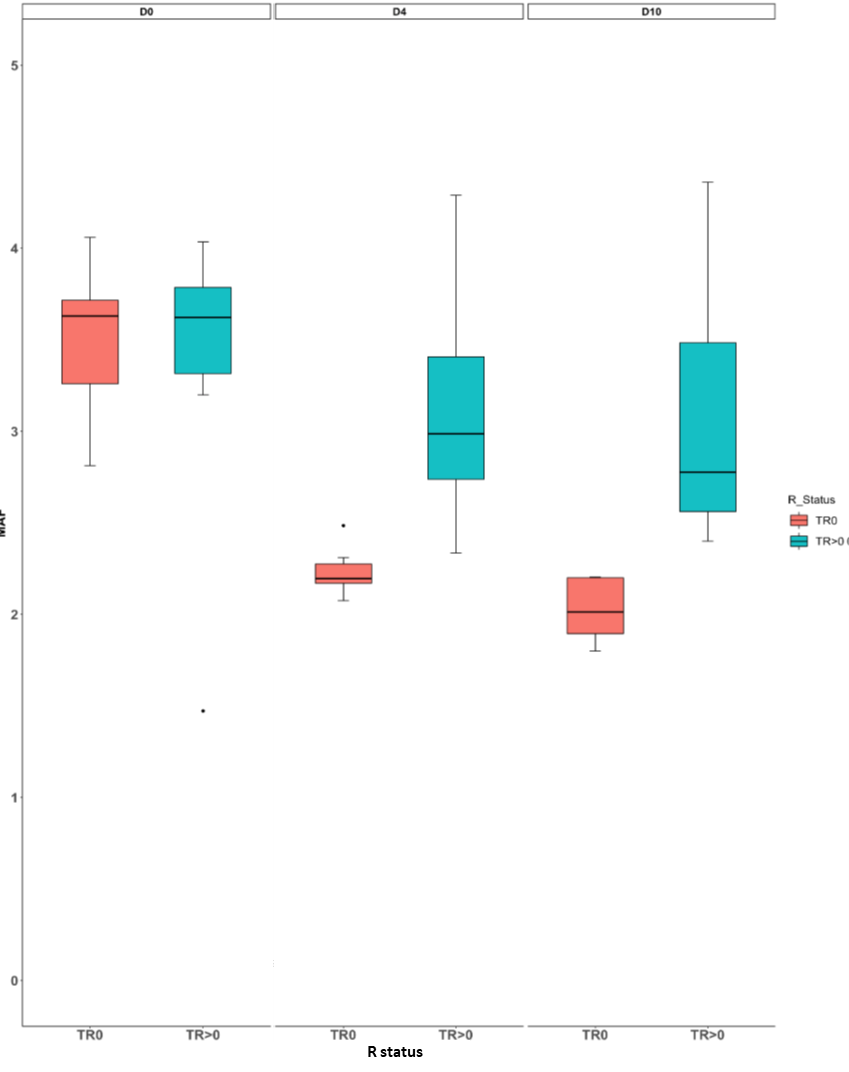


Δlog ascites HE4

p=0.000

p=0.000

n.s.

Supplement 1 Figure D: **Ascites HE4 in dependency of tumor burden** (debulking status after surgery); R status- residual disease after surgery; D0 baseline; D4 day 4 after surgery; D10 day 10 after surgery; p-values report comparison between TR0 and TR>0; n.s.- not significant
