# Supplementary material for: Cell-free tumor DNA, CA125 and HE4 for the objective assessment of tumor burden in patients with advanced high-grade serous ovarian cancer
Source: PLoS One. 2022 Feb 7;17(2):e0262770. doi: 10.1371/journal.pone.0262770 (PMC8820624; doi:10.1371/journal.pone.0262770)
Supplement: S2 Fig — A CA125 in Serum; B HE4 in Serum; C CA125 in ascites; D HE4 in ascites. (DOCX) [file pone.0262770.s002.docx]

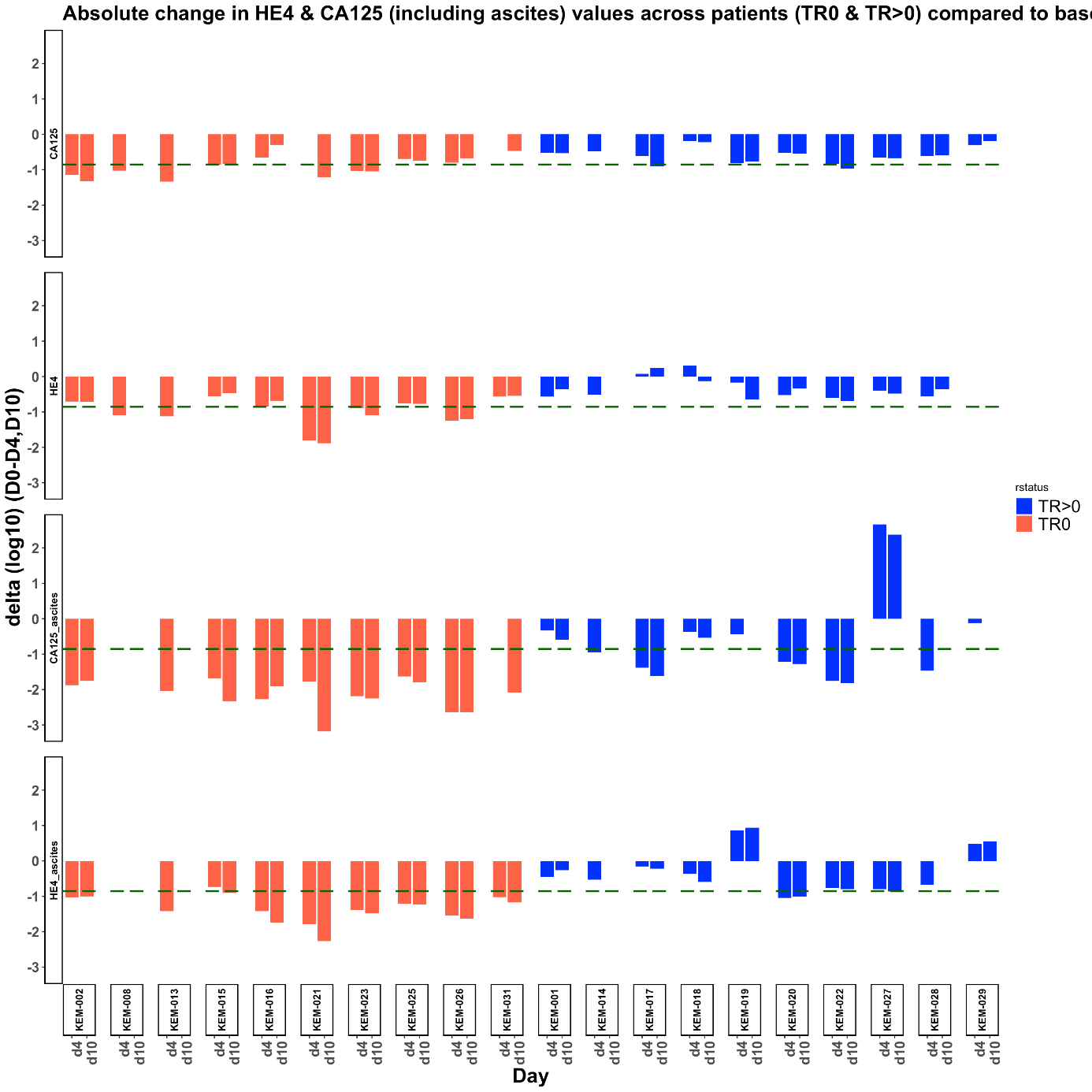


D

B

C

A

Supplement 2 Figure: Individual absolute change of serum and ascites CA125 and HE4 across all patients with complete (TR0, orange)) and incomplete resection (TR>0, blue) between baseline and day 4(d4) and day10 (d10). A CA125 in Serum; B HE4 in Serum; C CA125 in ascites; D HE4 in ascites
