# Supplementary material for: Cell-free tumor DNA, CA125 and HE4 for the objective assessment of tumor burden in patients with advanced high-grade serous ovarian cancer
Source: PLoS One. 2022 Feb 7;17(2):e0262770. doi: 10.1371/journal.pone.0262770 (PMC8820624; doi:10.1371/journal.pone.0262770)
Supplement: S3 Fig — a Number of packed blood units given between atients with TR0 and TR>0. b: Correlation between packed blood units and TP53 mutations depending TR0 and TR>0 at the post-surgical days. (DOCX) [file pone.0262770.s003.docx]

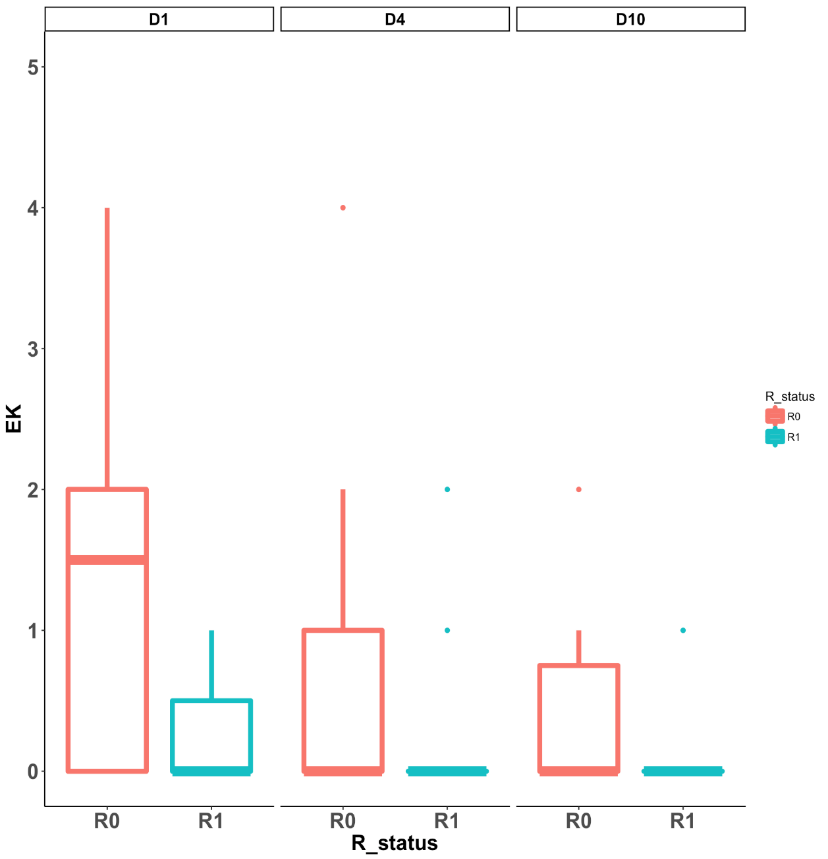


p=0.26

p=0.31

p=0.78

Supplement 3a Figure: **Number of packed blood** units given between atients with TR0 and TR>0


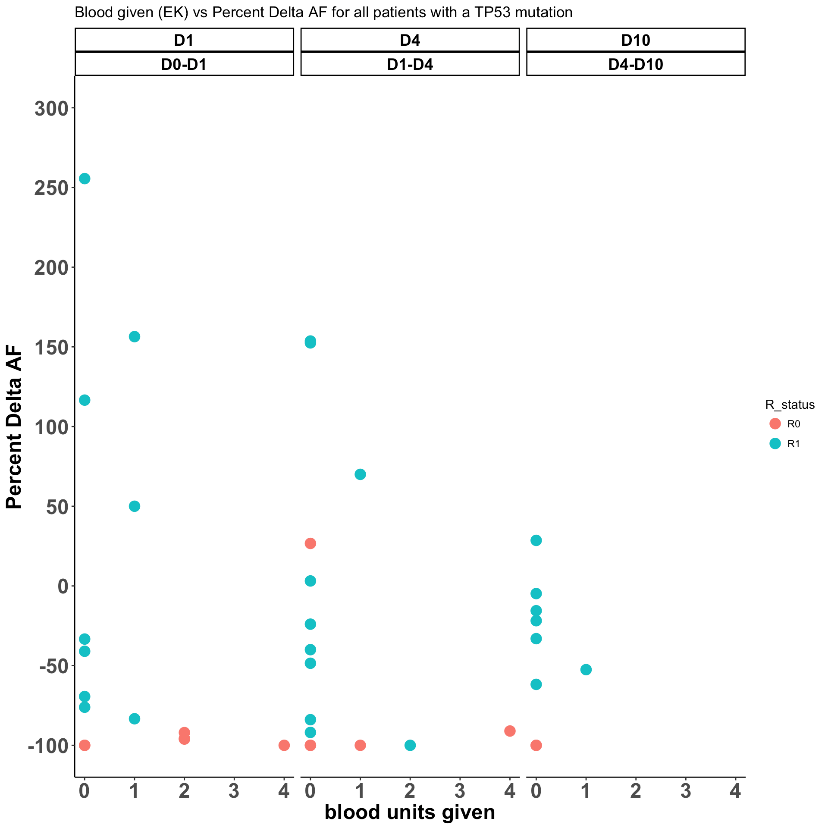


Supplement 3b Figure: **Correlation between packed blood units and TP53** mutations depending TR0 and TR>0 at the post-surgical days.
