# Supplementary material for: Cell-free tumor DNA, CA125 and HE4 for the objective assessment of tumor burden in patients with advanced high-grade serous ovarian cancer
Source: PLoS One. 2022 Feb 7;17(2):e0262770. doi: 10.1371/journal.pone.0262770 (PMC8820624; doi:10.1371/journal.pone.0262770)
Supplement: S1 Table — a: Genes included on the panel for ct-DNA analyses; b: Amplicons targeted for sequencing of the genes included on the panel for ct-DNA analyses. (DOCX) [file pone.0262770.s004.docx]

Supplement 1a Table: **Genes included on the panel for ct-DNA analyses**

Supplement 1b Table: Amplicons targeted for sequencing of the genes included on the panel for ct-DNA analyses
