# Supplementary material for: Cell-free tumor DNA, CA125 and HE4 for the objective assessment of tumor burden in patients with advanced high-grade serous ovarian cancer
Source: PLoS One. 2022 Feb 7;17(2):e0262770. doi: 10.1371/journal.pone.0262770 (PMC8820624; doi:10.1371/journal.pone.0262770)
Supplement: S2 Table — (DOCX) [file pone.0262770.s005.docx]

Supplement 2 Table: Mutations found in tumor genome and at least one corresponding ct-DNA samples of each patient; yellow-coloured lines indicate private tissue- and blue-cloured lines indicate private ct-DNA mutations.
